# Force-induced changes of α-catenin conformation stabilize vascular junctions independently of vinculin

**DOI:** 10.1242/jcs.259012

**Published:** 2021-12-22

**Authors:** Cao Nguyen Duong, Randy Brückner, Martina Schmitt, Astrid F. Nottebaum, Laura J. Braun, Marika Meyer zu Brickwedde, Ute Ipe, Hermann vom Bruch, Hans R. Schöler, Giuseppe Trapani, Britta Trappmann, Mirsana P. Ebrahimkutty, Stephan Huveneers, Johan de Rooij, Noboru Ishiyama, Mitsuhiko Ikura, Dietmar Vestweber

**Affiliations:** 1 Department of Vascular Cell Biology, Max Planck Institute for Molecular Biomedicine, D-48149 Münster, Germany; 2 Department of Cell and Developmental Biology, Max Planck Institute for Molecular Biomedicine, D-48149 Münster, Germany; 3 Bioactive Materials Laboratory, Max Planck Institute for Molecular Biomedicine, D-48149 Münster, Germany; 4 Institute of Medical Physics and Biophysics, University of Muenster, Muenster 48149, Germany; 5 Amsterdam University Medical Center, Location AMC, University of Amsterdam, Amsterdam, The Netherlands; 6 Center for Molecular Medicine, University Medical Center Utrecht, 3584 CG Utrecht, The Netherlands; 7 Princess Margaret Cancer Centre, University Health Network, Toronto, ON M5G 1L7, Canada

**Keywords:** Cadherin, Mechanobiology, Cell adhesion, Endothelial cell junctions, Vascular biology

## Abstract

Cadherin-mediated cell adhesion requires anchoring via the β-catenin–α-catenin complex to the actin cytoskeleton, yet, α-catenin only binds F-actin weakly. A covalent fusion of VE-cadherin to α-catenin enhances actin anchorage in endothelial cells and strongly stabilizes endothelial junctions *in vivo*, blocking inflammatory responses. Here, we have analyzed the underlying mechanism. We found that VE-cadherin–α-catenin constitutively recruits the actin adaptor vinculin. However, removal of the vinculin-binding region of α-catenin did not impair the ability of VE-cadherin–α-catenin to enhance junction integrity. Searching for an alternative explanation for the junction-stabilizing mechanism, we found that an antibody-defined epitope, normally buried in a short α1-helix of the actin-binding domain (ABD) of α-catenin, is openly displayed in junctional VE-cadherin–α-catenin chimera. We found that this epitope became exposed in normal α-catenin upon triggering thrombin-induced tension across the VE-cadherin complex. These results suggest that the VE-cadherin–α-catenin chimera stabilizes endothelial junctions due to conformational changes in the ABD of α-catenin that support constitutive strong binding to actin.

## Abstract (precis)

**Summary:** There are novel antibody epitopes at the actin-binding domain of α-catenin that correlate with high affinity binding and are exposed in junction-stabilizing VE-cadherin–α-catenin fusion proteins.

## INTRODUCTION

The dynamic control of endothelial cell junctions is central for the regulation of the vascular barrier, the control of vascular leaks and leukocyte entry into tissue at sites of inflammation. The integrity of endothelial junctions strongly relies on the adhesive function of VE-cadherin (also known as CDH5) and its mechanical coupling with the actin cytoskeleton. As for other cadherins, actin coupling is achieved by the binding of VE-cadherin to β-catenin (encoded by *CTNNB1*), which in turn binds to α-catenin (herein referring to αE-catenin, encoded by *CTNNA1*, unless otherwise stated), which links the complex to F-actin (Nagafuchi and Takeichi, 1988, 1989; Ozawa et al., 1989, 1990). We have previously replaced this triple complex in mice by a chimera containing VE-cadherin directly fused to α-catenin (Schulte et al., 2011). In these mice, the induction of vascular permeability by various inflammatory mediators was completely blocked, and leukocyte extravasation in several tissues was strongly impaired (Schulte et al., 2011). These findings supported the concept that leukocytes transmigrate through endothelial barriers mainly via the trans-junctional route whereas the transcellular pathway is of minor importance.

Probing the mechanism of junction stabilization, it was found that VE-cadherin–α-catenin (hereafter denoted VEC-αC) was recruited more efficiently to F-actin, given that detergent extractability as well as membrane mobility of the chimera were severely reduced in comparison to normal VE-cadherin (Schulte et al., 2011). However, why anchoring to the actin cytoskeleton was increased remained unclear.

The essential role of α-catenin for the anchoring of cadherins to the actin cytoskeleton is complex and relies on direct interactions as well as on adaptor proteins that can link α-catenin to F-actin. α-Catenin contains three major domains, the N-terminal β-catenin-binding domain, the modulation (M) domain (consisting of domains M1 to M3) and the actin-binding domain (ABD). Direct binding of α-catenin to F-actin is based on catch bond behavior (i.e. the interaction is strengthened under mechanical load; Buckley et al., 2014). This is based on force-induced conformational changes of the α-catenin ABD, which improves its binding to F-actin. The analysis of various mutants of α-catenin has allowed the structural changes of the ABD linked to low- and high-affinity binding to F-actin to be determined (Ishiyama et al., 2018; Xu et al., 2020). Based on steered molecular dynamics (SMD) simulations, a short N-terminal α1-helix of the ABD (residues 669–675, in αE-catenin) becomes unfolded upon simulating pulling forces (Ishiyama et al., 2018). Since mutations causing unfolding of this helix in recombinant forms of the ABD enhanced its binding to F-actin, it was suggested that force-dependent unfolding of this structural element is important for catch bonding (Ishiyama et al., 2018). These results were confirmed and extended to further important conformational changes by analyzing the cryo-electron microscopy structures of the actin-bound form of the α-catenin ABD (Mei et al., 2020; Xu et al., 2020).

The force threshold of ∼5 pN for catch bonding of α-catenin (Buckley et al., 2014) is in the same range as the force needed to allow binding of the actin adaptor vinculin to α-catenin (Yao et al., 2014). Vinculin binds to the M1 domain of α-catenin, which is masked under zero force conditions and requires force-induced unmasking for binding (Choi et al., 2012; Ishiyama et al., 2013; le Duc et al., 2010; Watabe-Uchida et al., 1998; Yonemura et al., 2010). Importantly, it has been shown that expression of αE-catenin mutated in its vinculin-binding site fails to rescue cadherin-based adhesion strength in cells lacking αE-catenin (Thomas et al., 2013). Furthermore, vinculin was reported to protect endothelial junctions from opening during their force-dependent remodeling (Huveneers et al., 2012). Thus, mechanical load could enhance cadherin catenin anchoring to the actin cytoskeleton in multiple ways.

Here, we have analyzed why the VEC-αC chimera stabilizes endothelial junctions and interacts more stably with the actin cytoskeleton. We found that VEC-αC indeed recruited vinculin constitutively to junctions, yet this was dispensable for endothelial junction stabilization of the mouse vasculature *in vivo*. Instead, a tension-dependent conformational epitope of the ABD of α-catenin is constitutively exposed in VEC-αC at cellular junctions. These results suggest, that conformational changes of the ABD, but not of the M domain of α-catenin, are the reason for the junction-stabilizing effect of the VEC-αC chimera.

## RESULTS

### Vinculin binds constitutively to VEC-αC

We have previously generated knock-in mice with highly stabilized endothelial junctions by inserting a VEC-αC fusion construct into the VE-cadherin genomic locus, thereby expressing it under the endogenous VE-cadherin promotor and replacing endogenous VE-cadherin (Schulte et al., 2011). Binding of the VEC-αC chimera to β-catenin was avoided by deleting 75 amino acids at the C-terminus of VE-cadherin and 300 amino acids of the N-terminus of α-catenin, leaving most of the M domain and the complete ABD intact.

**Fig. 1.**
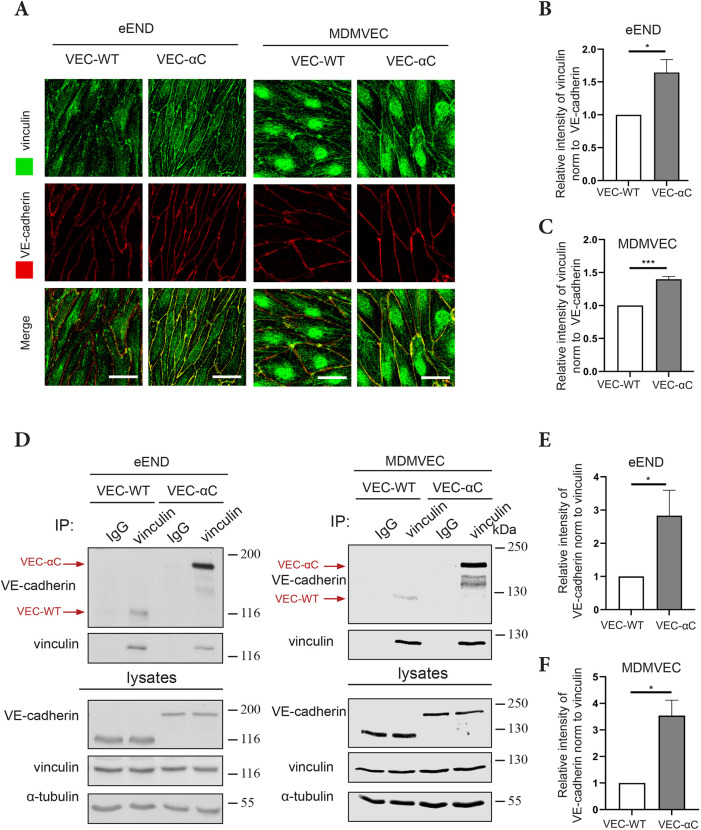
**VEC-αC strongly associates with vinculin.** (A) eEND cells or MDMVECs from VEC-WT and VEC-αC mice were fixed and stained for indicated antigens. Scale bars: 20 µm. (B,C) Quantification of vinculin signal intensities at cell contacts relative to VE-cadherin signal intensities as shown in A (*n*=3 independent experiments). (D) Vinculin was precipitated from cell lysates of eEND cells or MDMVECs using anti-vinculin antibodies or isotype-matched control antibodies. Immunoprecipitates or cell lysates were analyzed by SDS-PAGE and immunoblotted for VE-cadherin, vinculin or α-tubulin. Positions of molecular mass markers are indicated on the right. The lysate blots show 2% of input. (E,F) Quantification of VE-cadherin signal relative to vinculin signal intensities as shown in D (E, *n*=5; F, *n*=3 independent experiments). Results are shown as means±s.e.m. *P≤0.05; ***P≤0.001 (unpaired two-tailed Student's *t*-test).

Since detergent extractability and membrane mobility of VEC-αC were reduced in comparison to VE-cadherin (Schulte et al., 2011), we assumed that the chimera bound more efficiently to the actin cytoskeleton. To elucidate the molecular basis of this improved interaction, we tested whether the binding to the actin adaptor vinculin was increased. The binding site of vinculin in the M1 domain of α-catenin is usually inaccessible under zero force conditions and becomes unmasked upon exposure of the cadherin–catenin complex to force (Yonemura et al., 2010; Huveneers et al., 2012). To test, whether this interaction with vinculin is modified for VEC-αC, we compared the recruitment of vinculin to either normal VE-cadherin–catenin complexes or to VEC-αC in mouse endothelial cells. For this, we examined the presence of vinculin at junctions of mouse endothelioma cells (eEND) or alternatively primary isolated endothelial cells (MDMVECs) from the skin of either wild-type (WT) mice or VEC-αC knock-in mice. As shown in Fig. 1A–C, vinculin was clearly more abundant at endothelial cell junctions when cells expressed the VEC-αC chimera, and this was valid for endothelioma cells as well as for primary endothelial cells. In contrast, VE-cadherin staining was of similar intensity for normal VE-cadherin and VEC-αC.

We then tested the interactions with vinculin more directly by immunoprecipitating vinculin from lysates of eEND and MDMVECs of both genotypes and probing in immunoblots for the amount of either co-immunoprecipitated VE-cadherin or of VEC-αC. We found that VEC-αC was much more efficiently co-precipitated than normal VE-cadherin, independently of the type of endothelial cells (eEND or MDMVECs) that was analyzed (Fig. 1D–F). This is in line with the crystal structure of α-catenin (e.g. PDB 4IGG), according to which the VEC-αC chimera would lack the first α-helix of the M1 domain, which affects the formation of a four-helix bundle necessary to occlude the vinculin-binding domain (VBD) in an autoinhibitory conformation.

Collectively, these results suggest that the VBD is largely masked in WT α-catenin, but constitutively accessible in the VEC-αC chimera.

### Generation and characterization of VEC-αC-ΔVBD and VEC-αC-swapVBD knock-in mouse lines

**Fig. 2.**
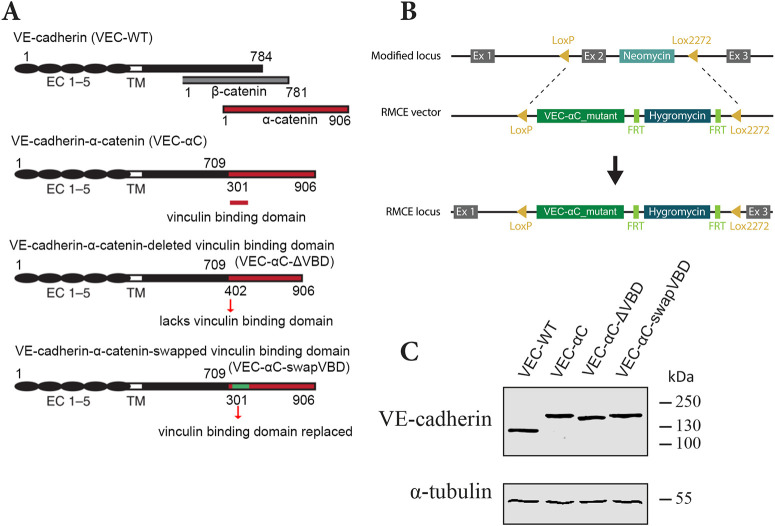
**Characterization of the VEC-αC-ΔVBD and VEC-αC-swapVBD constructs.** (A) Schematic illustration of VE-cadherin, β-catenin, α-catenin, and VEC-αC, VEC-αC-ΔVBD and VEC-αC-swapVBD fusion proteins. VEC-αC consists of a truncated form of VE-cadherin lacking the β-catenin-binding site (C-terminal 75 aa), which was fused to the C-terminal part (aa 301–906) of α-catenin. VEC-αC-ΔVBD and the VEC-αC-swapVBD are both unable to associate with vinculin and were constructed by modifying VEC–αC. The vinculin binding domain of α-catenin (aa 301–401) was deleted in VEC-αC-ΔVBD or was replaced by a homologous domain from mouse vinculin (green) in VEC-αC-swapVBD. Numbers refer to amino acid positions. (B) Targeting strategy for the generation of VEC–αC mutant knock-in mice by recombinase-mediated cassette exchange (RMCE). (C) MDMVECs were isolated from the four knock-in mouse lines (as indicated) and lysates were immunoblotted for VE-cadherin and α-tubulin (representative of *n*=2 independent experiments).

To test the relevance of constitutive binding of vinculin to VEC-αC for endothelial junction stabilization *in vivo*, we generated two equivalent knock-in mouse lines in which the VBD of VEC-αC was either deleted or modified, thereby preventing the association of VEC-αC with vinculin (Fig. 2A). In one case, most of the M1 domain (amino acid 301–401) containing the VBD was deleted (VEC-αC-ΔVBD). To avoid too drastic structural changes of the original VEC-αC chimera, an alternative construct was generated by swapping most of the M1 domain for the corresponding domain of vinculin (VEC-αC-swapVBD). Importantly, this vinculin domain is unable to bind to vinculin (Huveneers et al., 2012). The two mutated VEC-αC knock-in mouse lines were successfully established using recombinase-mediated cassette exchange (RMCE) technology (Schulte et al., 2011), replacing endogenous VE-cadherin by the VEC-αC mutants (Fig. 2B). Homozygous mutant pups were borne for each of the two new mouse lines at sub-Mendelian levels, pointing to partial embryonic lethality (Table S1), similar to what was found for the original VEC-αC mice (Schulte et al., 2011).

To characterize the modified VEC-αC mutants in detail, primary ECs were isolated from the skin of both mouse lines and lysates of these cultured cells were subjected to western blotting for VE-cadherin. As shown in Fig. 2C, both mutants completely replaced endogenous VE-cadherin and showed the expected size on SDS-PAGE gels. Specifically, VEC-αC-swapVBD displayed the same apparent molecular mass as VEC-αC, whereas VEC-αC-ΔVBD was slightly smaller.

**Fig. 3.**
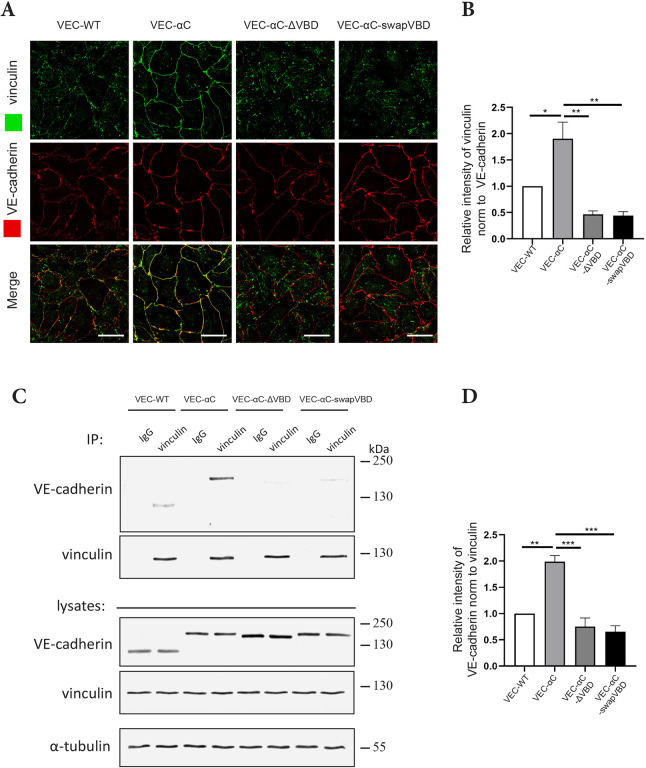
**Impaired vinculin recruitment by VEC-αC-ΔVBD and VEC-αC-swapVBD.** (A) MDMVECs from the indicated mouse lines were fixed and stained for the indicated antigens. Scale bars: 25 µm. (B) Quantification of vinculin signal intensities at cell contacts relative to VE-cadherin signal intensities as shown in A (*n*=3 independent experiments). (C) Anti-vinculin and control (IgG) immunoprecipitates of MDMVECs from the four indicated knock-in mouse lines (IP) or complete cell lysates (lysates; 2% of input) were immunoblotted for VE-cadherin, vinculin or α-tubulin. Positions of molecular mass markers are indicated on the right. (D) Quantification of VE-cadherin signal relative to vinculin signal intensities as shown in C (*n*=3 independent experiments). Results are shown as means±s.e.m. *P≤0.05; **P≤0.01; ***P≤0.001 (one-way ANOVA with Tukey's multiple comparisons test).

Next, we compared the junctional expression of all three VEC-αC versions and their ability to recruit vinculin. Immunostaining of confluent MDMVECs isolated from WT mice and the three knock-in mouse lines revealed, that the expression level of each VEC-αC chimeric protein at junctions was similar to that of VE-cadherin, and that each of the three chimeras caused some linearization of cell contacts in comparison to what was seen with VE-cadherin (Fig. 3A). Vinculin was much more strongly recruited to junctions in VEC-αC MDMVECs when compared to VE-cadherin, whereas no junctional vinculin recruitment was observed in VEC-αC-ΔVBD or VEC-αC-swapVBD MDMVECs (Fig. 3A,B). In line with this, we found in immunoblots that neither VEC-αC-ΔVBD nor VEC-αC-swapVBD could be efficiently co-immunoprecipitated with anti-vinculin antibodies from these cultured MDMVECs, whereas VE-cadherin was weakly and VEC-αC was strongly co-precipitated (Fig. 3C,D). Thus, the VEC-αC-ΔVBD and the VEC-αC-swapVBD chimeras are properly expressed at junctions, but lose the ability to recruit vinculin.

### Constitutive binding of VEC-αC to vinculin is irrelevant for junction stabilization in VEC-αC mice

**Fig. 4.**
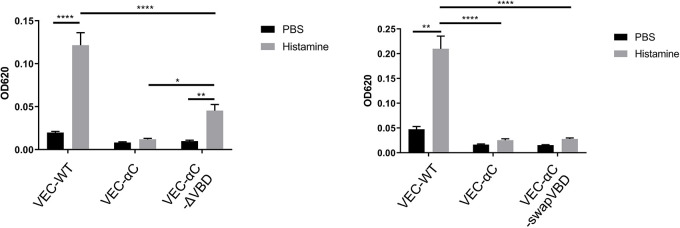
**Induction of vascular permeability is completely blocked in VEC-αC and in VEC-αC-swapVBD, but only partially blocked in VEC-αC-ΔVBD knock-in mice.** VEC-WT, VEC-αC, and either VEC-αC-ΔVBD mice (left graph) or VEC-αC-swapVBD mice (right graph) were intravenously injected with Evans Blue, followed by intradermal injection of histamine or PBS after 15 min. At 30 min after injection, skin areas were excised and the dye was extracted and quantified. Data are pooled from at least two independent experiments with seven to ten mice per group. Results are shown as means±s.e.m. *P≤0.05; **P≤0.01; ****P≤0.0001 (two-way ANOVA with Tukey's multiple comparisons test). OD620, optical density at 620 nm.

Next, we compared the ability of the different VEC-αC constructs to block the induction of vascular permeability *in vivo*. To this end, we performed Miles assays with each of the four mouse lines (VEC-WT, VEC-αC, VEC-αC-ΔVBD and VEC-αC-swapVBD) by injecting Evans Blue intravenously and stimulating intradermally with histamine. After 30 min, permeability was measured by extraction of the leaked dye from the excised skin area with formamide. In line with our previous study (Schulte et al., 2011), the strong histamine-induced increase of permeability that we observed in VEC-WT mice was completely blocked in VEC-αC mice (Fig. 4). Interestingly, VEC-αC-ΔVBD blocked the induction of vascular permeability only partially, reducing the increase observed in VEC-WT mice by 63% (Fig. 4, left panel). In contrast, VEC-αC-swapVBD blocked the induction of vascular permeability as completely as did VEC-αC (Fig. 4, right panel). Thus, deleting the VBD of VEC-αC partially impaired the junction stabilizing effect of the chimera, whereas replacing this domain by a homologous, non-vinculin-binding domain left the junction-stabilizing effect intact. This excludes a role for vinculin in the junction stabilizing effect of VEC-αC *in vivo*.

**Fig. 5.**
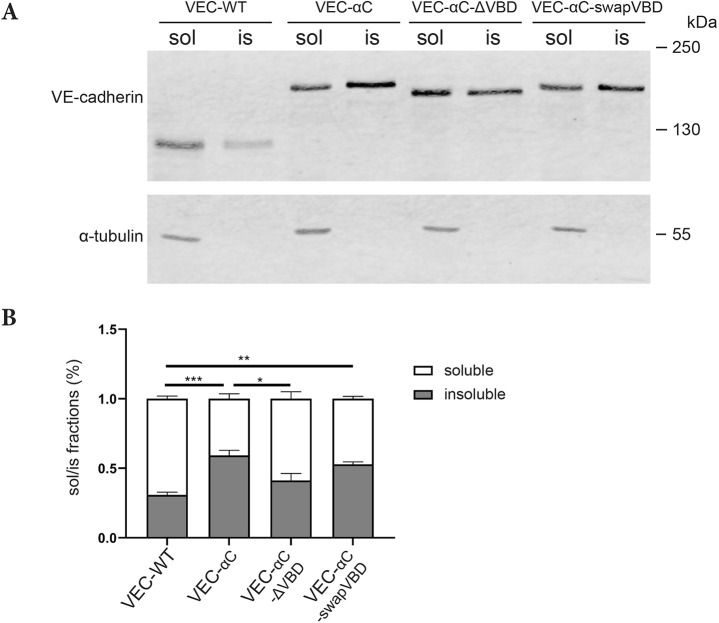
**Deleting the VBD in VEC-αC, but not replacing it enhances detergent extractability.** (A) To separate the Triton-soluble (sol) from the Triton-insoluble (is) fraction of the various forms of VE-cadherin, confluent MDMVECs were incubated with Triton buffer for 10 min (soluble fraction), followed by lysing the residual monolayers in a harsh SDS-containing lysis buffer (insoluble fraction). Both fractions were subjected to western blotting for the indicated antigens. (B) Quantification of soluble/insoluble fractions from four independent experiments. Results are shown as mean±s.e.m. *P≤0.05; **P≤0.01; ***P≤0.001 (one-way ANOVA with Tukey's multiple comparisons test).

The unexpected differences between the effects of VEC-αC-ΔVBD and VEC-αC-swapVBD on junction stabilization prompted the idea that the complete deletion of aa 301–401 in the VEC-αC-ΔVBD may have caused allosteric structural changes that reduced actin binding in a vinculin-independent way, whereas the more subtle replacement of this domain by a non-vinculin-binding domain left the strong constitutive binding to actin intact. To test this, we compared detergent extractability of the different constructs from primary MDMVECs. Upon extraction of MDMVEC monolayers with a mild detergent buffer (soluble fraction), insoluble residual material was re-extracted with an SDS-containing buffer (insoluble fraction) and the two fractions were analyzed by immunoblotting. As shown in Fig. 5, 31% of VE-cadherin, but 59% of VEC-αC, was found in the insoluble fraction. In contrast, only 41% of VEC-αC-ΔVBD was present in the insoluble fraction versus 53% of the VEC-αC-swapVBD chimera. Thus, VEC-αC-ΔVBD is less resistant to detergent extraction than VEC-αC, in line with its less efficient junction stabilization. In contrast, VEC-αC-swapVBD shows similar resistance to detergent extractability to that of VEC-αC and is as efficient in junction stabilization. We therefore conclude that both junction stabilization and resistance to detergent extractability of VEC-αC do not require vinculin anchorage.

### Changes in the conformation of the ABD of α-catenin in VEC-αC

**Fig. 6.**
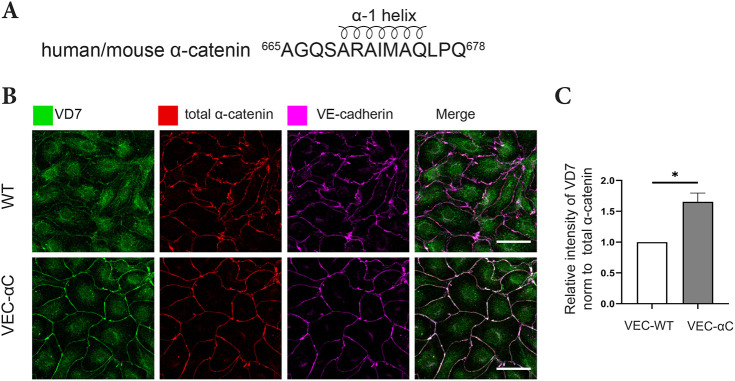
**Constitutive unfolding of the α1-helix of α-catenin in junctional VEC-αC.** (A) Peptide sequence of α-catenin used to generate the VD7 antibodies. The part covering the α1-helix is indicated. (B) Confluent MDMVECs were fixed, permeabilized, and stained with VD7, total anti-α-catenin or anti-VE-cadherin antibodies. Scale bars: 30 μm. (C) Quantification of VD7 signal intensities at cell contacts relative to total α-catenin signal intensities as shown in B (*n*=3 independent experiments). Results are shown as means±s.e.m. *P≤0.05 (unpaired two-tailed Student's *t*-test).

Next, we wanted to determine whether conformational changes in the ABD of VEC-αC could be found, which might be relevant for its enhanced interaction with the actin cytoskeleton in endothelial cells. Based on SMD simulations of αN-catenin, it was recently suggested that a short N-terminal α1-helix of the ABD (in αE-catenin residues 669–675) unfolds upon simulating pulling forces (Ishiyama et al., 2018). Furthermore, inserting a mutation in this helix that impaired its folding, resulted in a recombinant form of the ABD, which showed enhanced binding to F-actin (Ishiyama et al., 2018). These results suggested that catch bonding of α-catenin to F-actin may be accompanied by unfolding of the α1-helix. Based on this concept, we speculated that the α1-helix might be constitutively unfolded in VEC-αC, explaining enhanced F-actin binding of the chimera in cells. To test this, we generated a rabbit polyclonal antibody (VD7) against a peptide covering amino acids 665 to 678 of human and mouse αE-catenin, which contained the 7 amino acids of the short α1-helix (Fig. 6A). Antibodies from this serum specifically recognize α-catenin in western blots (Fig. S1).

Using these antibodies, we stained cultured primary MDMVECs isolated from VEC-WT or VEC-αC mice. The same cells were co-stained with antibodies against VE-cadherin and antibodies that recognized total α-catenin independent of its conformation. As shown in Fig. 6B, the staining intensity for VE-cadherin and α-catenin was similar in both types of cells. In contrast, staining with VD7 was much stronger for VEC-αC-expressing cells than for VEC-WT-expressing cells (Fig. 6B). Quantification of VD7 signal intensities at cell contacts relative to total α-catenin signal intensities showed a significant increase in the VD7 staining in VEC-αC cells compared to VEC-WT cells (Fig. 6C). These results reveal that the epitopes recognized by the VD7 antibodies are much more accessible in VEC-αC than in normal α-catenin recruited to endothelial junctions. To rule out that staining might have been based on potential epitopes adjacent to the 7-amino-acid helix, we affinity purified antibodies from the serum with a peptide lacking the amino acids adjacent to this helix (Fig. S2A). With these antibodies, we again found strongly enhanced staining at junctions of cells expressing VEC-αC compared to that in those expressing VEC-WT (Fig. S2B,C). This indicates, that the α1-helix is unfolded in VEC-αC, which is in line with the concept that the α-catenin ABD in VEC-αC is conformationally modified and resembles the high-affinity conformation of ABD, which is formed when α-catenin is under tension.

### The antibody VD7 specifically recognizes α-catenin in a force-dependent manner

**Fig. 7.**
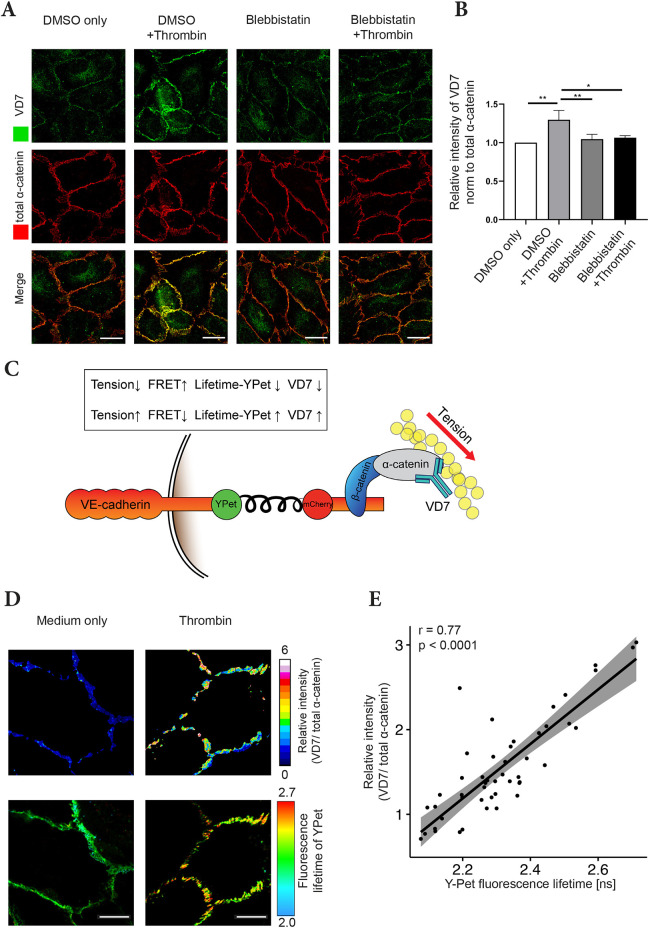
**The antibody VD7 recognizes α-catenin in a force-dependent manner.** (A) Confluent HUVEC monolayers were pre-treated with DMSO only or Blebbistatin for 30 min before treatment with medium only or thrombin for 10 min (as indicated). Cells were fixed, permeabilized and stained with VD7 and total α-catenin antibodies. (B) Quantification of VD7 signal intensities at cell contacts relative to total α-catenin signal intensities as shown in A (*n*=3 independent experiments). Results are shown as means±s.e.m. *P≤0.05; **P≤0.01 (one-way ANOVA with Tukey's multiple comparisons test). (C) Schematic representation of VE-cadherin tension sensor. (D) Confluent HUVEC monolayers expressing a VE-cadherin tension sensor were treated for 10 min with medium only or thrombin. Cells were fixed, permeabilized, and stained with VD7 and total α-catenin antibodies. The lifetime of YPet in VE-cadherin and the immunofluorescence intensity of VD7 and total α-catenin were acquired. (E) Scatter plot of the average fluorescence ratio (VD7/total α-catenin) and YPet lifetime of different cell–cell junctions. The points presented correspond to regions of interest segmented from a mixture of cells from a single representative experiment of four. Regression line with confidence interval of 95%. Spearman correlation (*r*) and *P*-value were calculated by a Spearman correlation test (*n*=48 values). Scale bars: 20 μm (A); 10 µm (D).

Next, we asked whether the VD7 antibody does indeed recognize α-catenin in a force-dependent manner. To this end, HUVECs were stimulated with thrombin in the presence or absence of the myosin II inhibitor Blebbistatin, and then fixed, permeabilized and stained with VD7 and control antibodies against total α-catenin. As shown in Fig. 7A and quantified in Fig. 7B, there was a significant increase in relative signal intensity of VD7 to total α-catenin staining in thrombin-treated HUVECs compared to that in control cells. Furthermore, pre-treatment with Blebbistatin abolished the increase in VD7 staining upon thrombin-treatment, showing that force created by the contractile actomyosin machinery is needed to unfold the α1-helix and allow staining with VD7 antibody.

To directly test whether tension across the VE-cadherin–catenin complex in cells coincides  with the unfolding of the α1-helix of α-catenin, we applied fluorescence lifetime imaging (FLIM) and made use of a recently generated VE-cadherin tension sensor (Arif et al., 2021). In this construct, a ferredoxin-like (FL) linker-based Förster resonance energy transfer (FRET) module was inserted between the p120-catenin (encoded by *CTNND1*) and the β-catenin-binding site of VE-cadherin. The FRET module contains YPet (donor) and mCherry (acceptor) separated by the elastic FL linker (Fig. 7C). Tension induces separation of YPet and mCherry, thereby increasing YPet lifetime. As the increase of VD7 staining in thrombin-treated HUVECs did not equally distribute to all junction areas, we wanted to test whether there is a pixel-by-pixel correlation between tension on VE-cadherin (YPet lifetime) and VD7 staining. To address this, HUVECs expressing the VE-cadherin tension sensor were treated with thrombin and stained with VD7 and for comparison with antibodies against total α-catenin. As shown in Fig. 7D and quantified in Fig. 7E, the ratio of pixel intensity for VD7/total α-catenin staining had a strong correlation with pixel lifetime of the YPet molecule. Thus, unfolding of the α1-helix, as monitored by an increase in VD7 epitope accessibility, coincided with enhanced tension across the VE-cadherin tension sensor.

Collectively, these results demonstrate that the VD7 antibody recognizes epitopes that are buried in the α1-helix of α-catenin and become accessible upon exposure to force.

### Analysis of direct binding of VEC-αC to F-actin

Since VD7 epitopes were strongly exposed in VEC-αC and these epitopes define catch bond-related conformational responses of α-catenin under mechanical load, we tested whether direct *in vitro* binding of a recombinant form of the cytoplasmic part of VEC-αC (cyto-VEC-αC) to F-actin would be enhanced in comparison to α-catenin. Monomeric α-catenin is known to dimerize, which strongly enhances binding to F-actin (Drees et al., 2005). Therefore, we subjected purified cyto-VEC-αC and α-catenin after His-tag based affinity chromatography to size exclusion chromatography, in order to separate monomers and dimers. The VEC-αC cytoplasmic tail eluted as a discrete peak, in agreement with the fact that the N-terminally located α-catenin dimerization site was deleted in this construct (Fig. S3A). Dimers and monomers of α-catenin were separated at different positions of the elution profile (Fig. S3A), in line with previous reports (Drees et al., 2005). Purity of the isolated proteins was monitored by SDS-PAGE and Coomassie staining (Fig. S3B).

Using an F-actin co-sedimentation assay, we compared the binding of cyto-VEC-αC to F-actin with the binding efficiency of monomeric and dimeric α-catenin. For these assays, each of the three protein preparations were incubated with F-actin for 30 min, followed by an ultra-speed sedimentation. The resulting supernatants (S) and pellets (P) were separated by SDS-PAGE and stained. For controls, ultra-speed sedimentations were performed in the absence of F-actin, to determine the amount of material that would unspecifically precipitate.

**Fig. 8.**
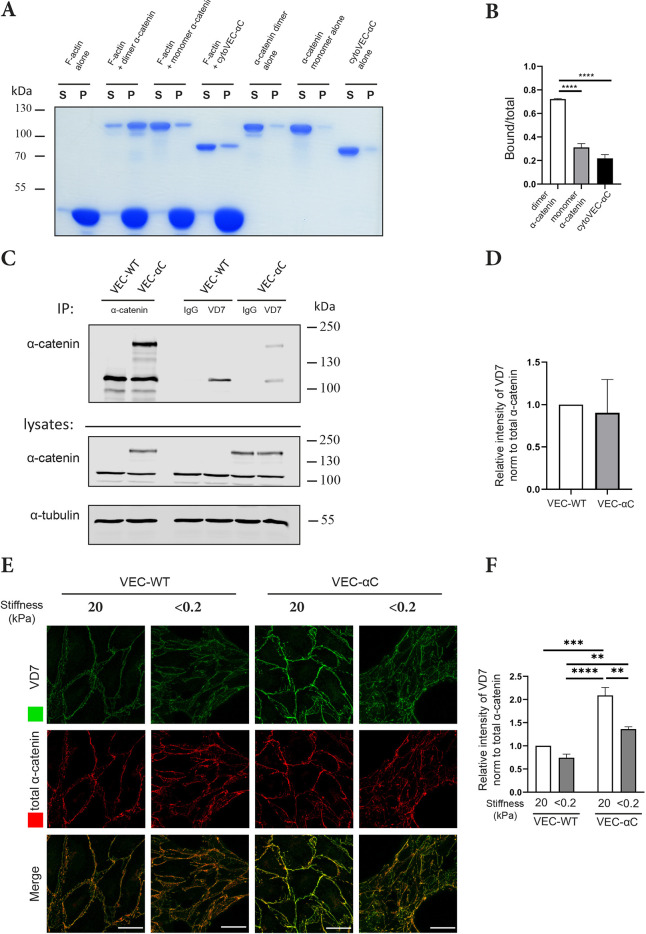
**Comparison of VEC-αC and α-catenin binding to F-actin.** (A) Sedimentation of dimeric and monomeric α-catenin and cytoVEC-αC in the presence and absence of F-actin. Supernatant containing the unbound protein (S) and pellet containing actin-bound protein (P) were analyzed by Coomassie-stained SDS-PAGE. (B) Quantification of actin-bound/ total fractions of the various recombinant forms of α-catenin as shown in A (*n*=3 independent experiments). (C) MDMVECs from VEC-WT or VEC-αC mice were subjected to immunoprecipitations with anti-total α-catenin, VD7 antibodies or control IgG (as indicated), followed by immunoblotting for α-catenin (upper panels); cell lysates were immunoblotted for the indicated antigens (bottom panels; 2% of input). The position of molecular mass markers are indicated on the right. (D) Quantification of VD7 signal relative to total α-catenin signal intensities as shown in C (*n*=4 independent experiments). (E) MDMVECs from VEC-WT or VEC-aC mice were grown on collagen-coated polyacrylamide gels of varying stiffness of <0.2 and 20 kPa for 72 h. Cells were fixed, permeabilized, and stained with VD7 and total α-catenin antibodies. (F) Quantification of VD7 signal intensities at cell contacts relative to total α-catenin signal intensities as shown in E (*n*=3 independent experiments). Results are shown as means±s.e.m. **P≤0.01; ***P≤0.001; ****P≤0.0001 [one-way ANOVA (B) or two-way ANOVA (F) with Tukey's multiple comparisons test, or the unpaired two-tailed Student's *t*-test (D)].

As expected, we found that most of α-catenin dimers bound to F-actin, whereas only a very small fraction of α-catenin monomers was co-sedimenting with F-actin (Fig. 8A,B). Unexpectedly, though, we found that soluble cyto-VEC-αC did not specifically co-sediment with actin any better than monomeric α-catenin. (Fig. 8A,B). Thus, binding of soluble cyto-VEC-αC to F-actin was not enhanced in comparison to monomeric α-catenin.

This unexpected result raised the question of whether the tension-dependent VD7 epitopes of α-catenin, which we found constitutively exposed in VEC-αC molecules at endothelial junctions, would perhaps not be exposed in soluble VEC-αC molecules. To this end, α-catenin antibodies against tension-independent epitopes and VD7 antibodies were used to immunoprecipitate α-catenin and VEC-αC from cell lysates of WT-VEC- or VEC-αC-expressing MDMVECs. Immunoprecipitates were analyzed by immunoblotting with anti-α-catenin antibodies. We found that VD7 antibodies recognized solubilized VEC-αC with similar efficiency to solubilized α-catenin (Fig. 8C). Quantification of the blot signals derived from material immunoprecipitated with VD7 or anti-total α-catenin antibodies revealed that the VD7 epitopes were not more strongly expressed on soluble VEC-αC than on soluble α-catenin molecules (Fig. 8D). The weak amounts of solubilized VEC-αC and α-catenin that could be precipitated with VD7 antibodies might be due to some antibodies recognizing epitopes at the very N-terminus or C-terminus of the peptide used as immunogen, which are not located within the α1-helix and might therefore be accessible in α-catenin independently of tension.

Collectively, these results suggest that the α1-helix is not constitutively unfolded in soluble VEC-αC. In contrast, we found that the soluble α-catenin dimer was much more efficiently immunoprecipitated with the VD7 antibody than monomeric α-catenin (Fig. S4). This shows that the VD7 epitope is clearly exposed when the ABD of α-catenin is in a high-affinity conformation. To test whether exposure of the VD7 epitope in VEC-αC at junctions requires force, we grew MDMVECs from VEC-WT or VEC-αC mice on substrate with either high stiffness (20 kPa) or low stiffness (<0.2 kPa), and stained cells with VD7 or anti-total α-catenin antibodies. As shown in Fig. 8E,F, staining of VEC-αC with VD7 was strongly reduced for cells grown on low stiffness substrates. Since stiffness of cell support is needed for the generation of tension in cells, this result shows that exposure of the VD7 epitope does indeed require tension even in the context of the VEC-αC fusion protein.

In summary, unfolding of the α1-helix in VEC-αC is not constitutive, but requires force. In contrast to normal monomeric α-catenin, unfolding of the helix in VEC-αC may require a lower force threshold for VEC-αC than for α-catenin. This would allow abundant exposure of the VD7 epitopes in VEC-αC located at endothelial junctions and might explain the formation of a conformation of the ABD in this chimeric protein that favors strong actin binding and thereby junction stabilization.

## DISCUSSION

Cadherins need to be linked by catenins to actin to support formation and stability of junctions between cells. Mechanical load enhances cadherin catenin anchorage to the actin cytoskeleton in at least two ways. First, force-induced conformational changes of the α-catenin ABD support catch bonding to actin. Second, forces of similar magnitude cause changes in the M domain of α-catenin, which exposes a cryptic vinculin-binding site and thereby an additional docking site for actin. Here, we show that both these changes are constitutively observed in a VEC-αC chimera that blocks the opening of endothelial cell junctions. Unexpectedly, the vinculin-binding site was irrelevant for the stabilization of vascular junctions, arguing that the conformational changes of the ABD were sufficient to render endothelial junctions irresponsive to permeability-inducing mechanisms. These conformational changes could be monitored in endothelial cells with the help of an antibody that recognizes a tension-sensitive epitope that underlies catch bonding of α-catenin to actin in the ABD. Our results suggest that constitutive catch bonding of α-catenin to actin is able to irreversibly stabilize endothelial junctions *in vivo*.

Conformational changes of the ABD of α-catenin induced by force were previously analyzed by equilibrium and constant force SMD. This revealed unfolding of the very short α1-helix close to the N-terminus of the ABD, which was linked to allosteric control of the dynamics of actin-binding residue V796 (Ishiyama et al., 2018). Introducing an unfolding mutation (H1) into this helix enhanced the binding of α-catenin ABD to F-actin in sedimentation assays. Similarly mutated full-length α-catenin-H1, when expressed in epithelial cell monolayers, rendered them much more resistant to mechanical disruption. In addition, epithelial sheet migration of these cells was reduced, suggesting impaired adherens junction dynamics. Finally, an analogous mutant of *Drosophila* α-catenin was not able to rescue embryonic lethality of zygotic-null mutants for α-catenin, again arguing that impaired dynamics of α-catenin actin interactions interferes with adherens junction function in developing epithelia (Ishiyama et al., 2018).

In the light of these previous results, it is revealing that we could indeed monitor tension-induced unfolding of the α1-helix in the ABD of α-catenin in thrombin-stimulated intact cells by staining of endothelial junctions with novel antibodies against tension specific epitopes. This confirms, in intact cells, the SMD modeling studies mentioned above. These results are also in agreement with recent cryoelectron microscopy studies (Mei et al., 2020; Xu et al., 2020), noting in the Xu et al. study, that although dissociation of the α2 helix was described as the major determinant of stable binding to actin, the α1-helix was suggested to be relevant for tuning the stability and force response of the ABD of α-catenin (Xu et al., 2020).

The functional consequences of directly mutating and unfolding the α1-helix (in α-Ecat-H1) for the dynamics and behavior of epithelial adherens junctions show some similarities with the effects caused by VEC-αC. Enhanced actin binding of the α-catenin ABD containing the H1 mutation (Ishiyama et al., 2018) is in agreement with reduced detergent extractability and membrane mobility of VEC-αC (Schulte et al., 2011). In addition, mechanical stabilization of epithelial junctions by expressing αEcat-H1 is in agreement with the stabilization of endothelial junctions caused by VEC-αC. In *Drosophila*, this stabilization effect caused lethality for all embryos whereas replacement of VE-cadherin by VEC-αC in mice was lethal for 50% of the offspring, when bred on a mixed C57Bl6/129SV genetic background (Dartsch et al., 2014; Schulte et al., 2011). In general, development of the blood vasculature was normal in all mice, whereas lethality was linked with defects in the entry of hematopoetic progenitors from the circulation into the fetal liver and impaired development of lymphatic vessels. In adult mice, leukocyte extravasation from the blood vasculature and induction of vascular permeability in inflammatory settings was impaired. All these defects are likely linked to impaired plasticity of endothelial junctions. Similarly, defects in epithelial junction dynamics are presumably linked to embryonic lethality caused by αEcat-H1 (Ishiyama et al., 2018).

The fact that development of the blood vasculature was by and large not affected by VEC-αC, whereas αEcat-H1 caused more dramatic embryonic defects could be either due to more robust mechanisms that ensure plasticity of endothelial junctions versus epithelial junctions. Alternatively, it is possible that the binding of αEcat-H1 to actin is stronger and less plastic than VEC-αC–actin association. This is in line with *in vitro* binding results, which revealed that soluble αEcat-H1 bound efficiently to F-actin in *in vitro* sedimentation assays, whereas no such binding was observed for recombinant cyto-VEC-αC. A third explanation for the more dramatic embryonic defects caused by the αEcat-H1 mutant could be based on the higher propensity of this mutant to bundle F-actin, which could further reduce plasticity at cellular junctions (Ishiyama et al., 2018).

The failure of cyto-VEC-αC to co-sediment with F-actin was unexpected, given that VEC-αC constitutively exposes VD7 epitopes at endothelial junctions at levels that are seen for normal α-catenin only under conditions of enhanced tension. In agreement with this, VEC-αC is less efficiently extractable by detergent and shows reduced membrane mobility, arguing for efficient actin interactions in intact cells. Equally unexpected, we found that VD7 antibodies did not immunoprecipitate solubilized VEC-αC with higher efficiency than normal α-catenin. This suggests that unfolding of the α1-helix is seen in VEC-αC only in intact cells and is reversible upon solubilization, probably due to the absence of mechanical load. In contrast, the soluble α-catenin dimer, which binds with high affinity to F-actin in the absence of force, strongly exposed the VD7 epitope, as we detected in immunoprecipitations. Collectively, these results suggest that baseline tension at endothelial junctions is sufficient to unfold the α1-helix in VEC-αC, but not in normal α-catenin. In agreement with this, we found that exposition of the VD7 epitopes on VEC-αC was dependent on force, since it was much less detectable when cells were grown on low stiffness substrates. This suggests that catch bonding of VEC-αC requires a lower tension threshold than normal α-catenin. It is possible that this is due to the fact that the first α-helix of the MI domain of α-catenin (aa 276–292) is missing in the VEC-αC construct. Since this helix is involved in masking of the VBD in the MI domain, and force is needed for unmasking, one could speculate that the force that is normally consumed to unmask the VBD could now immediately affect the conformation of the ABD, which would then affect the α1 helix, making the VD7 epitopes accessible. Thus, baseline forces at junctions are more exclusively directed towards unfolding the ABD of the chimera lacking the buffering effects of other structural elements.

We had originally expected that the constitutive binding of vinculin to VEC-αC would contribute to the stabilizing effect of this chimera on endothelial junctions. This was based on the fact that vinculin is well documented to provide stability to cell substrate interactions at focal adhesions when tension rises (Carisey et al., 2013; Spanjaard and de Rooij, 2013). Similarly, it was found that vinculin binding to α-catenin is required for maintenance of proper adhesion strength of cell contacts between E-cadherin-expressing cells (Ladoux et al., 2015; Thomas et al., 2013). In cultured epithelial cells, the binding of vinculin to α-catenin is needed to promote efficient barrier formation in calcium switch assays (Twiss et al., 2012). And in endothelial cells, recruitment of vinculin to α-catenin was found to protect junctions from opening by thrombin-induced actomyosin-mediated pulling forces (Huveneers et al., 2012). In this context, it is remarkable that conformational changes of α-catenin in our VEC-αC chimera, which make endothelial junctions resistant to inflammation-induced opening, are independent of vinculin–α-catenin interactions. This suggests that structural changes in VEC-αC, which make the ABD more responsive to tension-induced catch bonding to actin override the need for vinculin-mediated actin anchorage.

At present, we cannot exclude that conformational changes of α-catenin in the VEC-αC chimera may also have consequences for interactions with other molecular components that could be relevant for junction stability. α-Catenin binds other actin-binding proteins, such as α-actinin (Nieset et al., 1997), formin-1 (Kobielak et al., 2004) and afadin (Pokutta et al., 2002), which all bind to sites within the central part of the molecule, and ZO-1 (Imamura et al., 1999; Itoh et al., 1997) and EPLIN, which bind to the C-terminal part (Abe and Takeichi, 2008). Whether binding of these proteins is also modulated by force-induced conformational changes of α-catenin and whether these interactions enhance junction stability will be important to analyze.

Cadherin–α-catenin chimeras have been analyzed before in various experimental settings, leading to the stabilization of cellular junctions (Bianchini et al., 2015; Desai et al., 2013; Imamura et al., 1999; Nagafuchi et al., 1994; Ozawa and Kemler, 1998; Pacquelet and Rørth, 2005; Sarpal et al., 2012; Thomas et al., 2013). In one of these studies (Bianchini et al., 2015), it was demonstrated that enhanced actin binding of an E-cadherin–α-catenin chimera was due to the dimerization of this protein via the N-terminal dimerization domain of α-catenin. We can exclude this mechanism for the VEC-αC chimera studied here, since the dimerization domain is not present in the truncated form of α-catenin in this construct.

In conclusion, we present here novel antibodies that allow monitoring of conformational changes of the ABD of α-catenin, which are induced by elevated mechanical load and are thought to be relevant for the tuning of catch bonding to actin, at endothelial junctions. These conformational changes were constitutively observed in junctional VEC-αC under conditions of baseline tension and are likely relevant for the enhanced anchoring of VEC-αC to junctional actin and for the stabilization of endothelial junctions against inflammation-induced opening. The second conformational change of α-catenin in VEC-αC, which caused constitutive binding of the actin-adaptor vinculin, was irrelevant for endothelial junction stability.

## MATERIALS AND METHODS

### VEC-αC chimeras

The VEC-αC-ΔVBD fusion construct was generated by removing the vinculin-binding domain (aa 301–401 of αE-catenin) from the original VEC-αC chimera (Schulte et al., 2011). Using the VEC-αC cDNA as a template, XhoI restriction sites were introduced behind the cDNA sequence encoding VE-cadherin aa 709 and before aa 402 of α-catenin. After XhoI digestion, the vector was re-ligated, generating two additional amino acids [Leu (CTC) and Glu (GAG)] between the VE-cadherin and α-catenin cDNAs.

To generate VEC-αC-swapVBD cDNA, the VEC-αC-ΔVBD cDNA was digested with XhoI and the XhoI flanked mouse vinculin cDNA (aa 514–606) (Addgene plasmid #20144; Lien et al., 2008) was inserted. Next, the QuikChange Lightning Site-directed Mutagenesis Kit (Agilent Technologies, CA, USA) was used to delete both XhoI restriction sites and insert base pairs encoding α-catenin aa 301–303 5′ of the vinculin cDNA. Thus, with the exception of replacing the cDNA encoding α-catenin aa 304–401 by cDNA for vinculin aa 514–606, the remaining sequence of the VEC-αC-swapVBD plasmid was identical to the original VEC-αC plasmid.

### Mice

VEC-WT and VEC-αC knock-in mice were described previously (Schulte et al., 2011). VEC-αC-ΔVBD or VEC-αC-swapVBD knock-in mice were generated by inserting the appropriate cDNAs (see above) into the VE-cadherin locus via RMCE (Schulte et al., 2011), thereby replacing expression of endogenous VE-cadherin. All mice used in this study were on the mixed 129SV×C57Bl/6 genetic background. All animal studies were approved by Landesamt fuer Natur, Umwelt und Verbraucherschutz Nordrhein-Westfalen, Germany.

### Cell culture

Primary ECs from skin (MDMVECs) of VEC-WT, VEC-αC, VEC-αC-ΔVBD or VEC-αC-swapVBD mice were isolated and cultured as previously described (Frye et al., 2015). Endothelioma cells established from VEC-WT or VEC-α-C mice were cultured as previously described (Schulte et al., 2011). Human umbilical vein endothelial cells (HUVECs; Lonza, MD, USA) were cultured in EBM-2 medium supplemented with EGM-2 SingleQuots (Lonza, MD, USA) at 37°C and 5% CO_2_. Prior to experiments, cells were authenticated and confirmed to be free of contamination.

### Antibodies and reagents

The following antibodies were used (IF, immunofluorescence; WB, western blotting; IP, immunoprecipitation).

VD7 directed against the entire α1-helix sequence of α-catenin and its flanking regions (AGQSARAIMAQLPQ) was obtained by immunization of rabbits and affinity purification using a described method (including an additional N-terminal cysteine for coupling) (Ebnet et al., 2000; IF, 1 µg/ml; WB, 0.5 µg/ml; IP, 5 µg/reaction). To obtain antibodies that only recognized the α1-helix part, antibodies were affinity purified from this serum on the peptide GGGSARAIMAQ coupled with a N-terminal cysteine to SulfoLink resin (Thermo Fisher Scientific, MA, USA) (IF, 1 µg/ml). Rabbit polyclonal antibodies VE-42 (rab pAb) against mouse VE-cadherin have been described previously (Broermann et al., 2011) (WB, 0.5 µg/ml).

The following antibodies were commercially obtained: goat pAb AF1002 (R&D Systems, MN, USA) against mouse VE-cadherin (IF, 3 µg/ml), mouse monoclonal antibodies (mAb) V284 (Merck, Darmstadt, Germany) (IP, 5 µg/reaction), mouse mAb hVIN-1 (Sigma-Aldrich, MO, USA) (WB, 2 µg/ml) and rabbit mAb EPR8185 (Abcam, Cambridge, UK) (IF, 1:100) against vinculin, mouse mAb G11 (Santa Cruz Biotechnology, CA, USA) (IF, 5 µg/ml; WB, 1 µg/ml) and mAb alpha-CAT-7A4 (WB, 0.5 µg/ml; IP, 5 µg/reaction) (Thermo Fisher Scientific, MA, USA) against α-catenin, isotype control Ab (Rat IgG1, Thermo Fisher Scientific, MA, USA), (IP: 5 µg/reaction), mAb against α-tubulin (B-5-1-2, Sigma-Aldrich, MO, USA) (WB, 1:5000). Alexa Fluor 405-, Alexa-Fluor 488-, Alexa Fluor 568-, Alexa Fluor 594- and Alexa Fluor 647-coupled secondary antibodies were purchased from Invitrogen CA, USA) (IF, 1:700). IRDye 680RD- and IRDye 800CW-coupled secondary antibodies were purchased from LI-COR Biosciences (Cambridge, UK) (WB, 1:10,000). All other secondary antibodies were purchased from Dianova (Hamburg, Germany) (WB, 1:10,000).

HUVECs were stimulated with 1 U/ml thrombin (Calbiochem, CA, USA) for 10 min at 37°C and 5% CO_2_. To inhibit myosin II, HUVECs were treated with 50 μM Blebbistatin (Sigma-Aldrich, MO, USA) for 30 min. DMSO was used as vehicle control.

### Immunoprecipitation and immunoblotting

MDMVECs or eEND cells were lysed at 4°C for 10 min in IP-lysis buffer [1% Nonidet P-40, 25 mM Tris-HCl pH 7.4, 100 mM NaCl, 10 mM MgCl_2_, 10% glycerol, and Complete EDTA-free proteinase inhibitor cocktail (Roche, Penzberg, Germany)]. Lysates were cleared by centrifugation at 20,817 ***g*** for 2 min before aliquots for direct blot analysis were set aside and aliquots for immunoprecipitation were incubated for 1 h at 4°C with 4.5 μm Dynabeads (Thermo Fisher Scientific, MA, USA) loaded with the respective antibodies. Immunocomplexes were washed five times with lysis buffer and dissolved in Laemmli sample buffer for standard western blot analysis.

To analyze binding of the VD7 antibody to α-catenin forms, ∼200 ng of purified α-catenin dimers or monomers were diluted in 1 ml IP buffer (0.2% Nonidet P-40, 25 mM Tris-HCl pH 7.4, 100 mM NaCl, 10 mM MgCl_2_, 10% glycerol, and 1 mM DTT). Aliquots for direct blot analysis were set aside and α-catenin IP was performed as described above.

### Generation of polyacrylamide hydrogels

Polyacrylamide hydrogels attached to 12 mm glass coverslips were fabricated following an established protocol (Tse and Engler, 2010) with slight modifications. In brief, aqueous solutions with two different concentrations of acrylamide/bis-acrylamide (3%/0.025% and 8%/0.264%; Bio-Rad) were prepared to yield hydrogels of varying stiffness (Young's moduli of <0.2 kPa and 20 kPa, respectively). To enable cell adhesion, collagen I was conjugated to the hydrogel surface using the heterobifunctional linker sulfo-SANPAH. A 1 mg/ml aqueous solution of sulfo-SANPAH (Sigma, MO, USA) was pipetted on the hydrogel surface, followed by irradiation with 365 nm UV light (intensity of 10 mW/cm^2^) for 1 min. Substrates were washed with PBS and incubated with a 50 μg/ml rat-tail collagen I (Corning, NY, USA) solution in PBS for 3 h at 37°C. Samples were washed with PBS prior to cell seeding. Young's moduli of the hydrogels were characterized using a nanoindenter (Piuma, Optics 11, The Netherlands).

### Separation of cellular protein pools according to detergent solubility

Cells were rinsed twice with ice-cold PBS before extraction buffer I (20 mM Tris-HCl pH 7.4, 2 mM CaCl_2_, 2 mM MgCl_2_, 150 mM NaCl, 0.5% Triton-X-100, Complete EDTA-free proteinase inhibitors) was added and incubated for 10 min on ice. Subsequently, the buffer was collected and centrifuged at 20,817 ***g*** for 10 min at 4°C and the supernatant was defined as the high-Triton-X soluble fraction. Remaining cellular material on the dish was gently washed twice with cold PBS before addition of extraction buffer II [50 mM Tris-HCl pH 7.4,150 mM NaCl, 1% NP- 40, 2 mM EDTA, 1% (w/v) sodium deoxycholate, 0.5% (w/v) SDS, 0.01 M NaPi, Complete–EDTA–free proteinase inhibitors] and scraping all residual material off the dish. Lysis was performed for 30 min at 4°C in a head-over-end shaker and lysates were centrifuged for 30 min at 20,817 ***g*** and 4°C. Supernatants with proteins of high and low detergent solubility were subjected to SDS-PAGE and western blot analysis.

### Immunofluorescence staining

MDMVECs, eEND cells or HUVECs were seeded on fibronectin-coated chamber slides (ibidi, Martinsried, Germany, μ-Slide 8 Well) and grown to confluence. Cells were washed with PBS, fixed with 4% PFA in PBS for 7 min at room temperature, and permeabilized using 0.5% Triton X-100 in PBS for 10 min at room temperature, followed by blocking with 3% BSA, 2% donkey-serum for 2 h and incubation with primary antibodies. Primary antibodies were detected with Alexa Fluor-coupled secondary antibodies. Fluorescence signals were detected using a confocal laser-scanning microscope (LSM 780/LSM 880) with online fingerprinting mode [ZEN 2.3 (Zeiss, Oberkochen, Germany)]. ImageJ/Fiji or Imaris (Bitplane, Zurich, Switzerland) software were used for image processing. To quantify VD7 intensity at cell–cell junctions, a colocalization channel between VD7 and total α-catenin was created, and fluorescence intensity of VD7 was measured over the colocalization channel and normalized to fluorescence intensity of total α-catenin.

### RNA-mediated interference

For interference with human α-catenin expression, the siRNA 5′-ACAUGGCAGAUGUCUACAAtt-3′ produced by Life Technologies (CA, USA) was used. For interference with human VE-cadherin expression, CDH5 siRNA by Life Technologies was used (5′-GGGUUUUUGCAUAAUAAGCTT-3′). AllStars negative control siRNA (Qiagen, Hilden, Germany, sequence not provided), which does not target any known mammalian gene, was used as control. Routinely, HUVECs were transfected with 40–80 nM siRNAs for 48–72 h using INTERFERin^®^ (Polyplus, Illkirch, France) according to manufacturer's guidelines.

### FLIM-FRET and fluorescence intensity analysis via the VE-cadherin tension sensor during thrombin stimulation

VE-cadherin-siRNA treated HUVECs were seeded on fibronectin coated ibidi 8-well µ-chamber slides with 3×10^4^ cells per chamber, followed by re-expression of the VE-cadherin tension sensor (VEC-FL) (Arif et al., 2021) for 24 h. Cells were stimulated with thrombin for 10 min, washed with DPBS and fixed with 4% PFA in PBS for 5 min at 37°C. For IF analysis, cell monolayers were permeabilized using 0.5% Triton X-100 in PBS for 10 min at room temperature and blocked with 3% BSA, 2% donkey serum for 2 h, followed by incubation with VD7 and total α-catenin antibodies. Donkey anti-mouse-IgG conjugated to Alexa Fluor 405 and donkey anti-rabbit-IgG conjugated to Alexa Fluor 647 secondary antibodies were used to detect primary antibodies. FLIM images were acquired with a 485 nm pulsed laser at 40 MHz using ZEN 2.3 (Zeiss, Oberkochen, Germany) with SymPhoTime 64 2.4 (PicoQuant, Berlin, Germany). Signal was detected using a MultiHarp 150 TCSPC unit filtered by a 520/35 bandpass filter. Prior to each measurement, a *z*-stack of the fluorescence signal of VD7 and total α-catenin was recorded using 405 and 633 nm constant lasers within the same field of view as the FLIM acquisition.

For the analysis of FLIM-FRET data, pixel-wise data fitting (SymPhoTime, Picoquant, Berlin, Germany) was used with subsequent bi-exponential tail-fitting. For fluorescence intensity images, ImageJ/Fiji was used to create a mask based on total α-catenin intensity signal. This mask was further segmented with the Watershed segmentation algorithm (ImageJ/Fiji) to create different regions of interest (ROIs). Next, the plugin ‘Ratio Plus’ was used to create a pixel-by-pixel ratio image of VD7 to total α-catenin intensity. Average ratio and VEC-FL lifetime of the automatically generated ROIs were used for correlation analysis in R software (Version 1.3.1093).

### *In vivo* vascular permeability assay in the skin

A modified Miles assay for the induction of vascular permeability in the skin was performed as described previously (Mamluk et al., 2005). 8–12-week-old female mice were used for this assay. Evans Blue dye (Sigma-Aldrich) was injected into the tail vein (100 μl of a 1% solution in PBS), and after 15 min, 50 μl PBS or 225 ng histamine in 50 μl PBS was injected intradermally into the shaved back skin. 30 min later, skin areas were excised and extracted with formamide for 5 days, and the concentration of the dye was measured at 620 nm with a spectrophotometer (Shimadzu, Kyoto, Japan).

### Recombinant protein expression and purification

cDNA encoding the mouse cytoplasmic tail of VEC-αC (cyto VEC-αC) [aa 623–784 of VE-cadherin fused to the C-terminal two-thirds of mouse α-catenin (aa 301–906)] was generated by PCR using the original VEC-αC cDNA as a template. The cDNA encoding cyto VEC-αC or cDNA encoding full-length mouse α-catenin were inserted into the pET21a vector (Sigma-Aldrich) to generate an in-frame fusion between mouse α-catenin and a C-terminal His_6_ tag. Recombinant fusion proteins were expressed in BL21 (DE3) Codon Plus *E. coli* cells, purified on HisTrap HP columns (Cytiva, MA, USA), and eluted with imidazole. Fusion proteins were further purified at 4°C using Superdex 200 gel filtration chromatography (ÄKTA, GE HealthCare, MA, USA) in 20 mM Tris-HCl pH 8.0, 150 mM NaCl and 2.5 mM DTT. Recombinant α-catenin containing a C-terminal His_6_ tag was expressed and purified similarly to cyto VEC-αC, and the monomer was separated from the dimer by two consecutive rounds of gel filtration chromatography on Superdex 200.

### F-actin co-sedimentation assay

The assay was performed using the Actin Binding Protein Spin-Down Biochem Kit (Cytoskeleton, CO, USA) according to the manufacturer's instructions with minor modifications. Rabbit muscle G-actin was incubated in 1× actin polymerization buffer (Cytoskeleton, CO, USA) for 1 h at room temperature to polymerize filaments. Gel filtered cytoplasmic tail of VEC-αC, as well as dimers and monomers of α-catenin were diluted to a final concentration of 1 µM in 1× reaction buffer (Cytoskeleton, CO, USA), with and without F-actin and incubated for 30 min at room temperature. Actin cushion buffer (Cytoskeleton, CO, USA) was pipetted into centrifuge tubes, then the reaction mixture was carefully loaded on top of the cushion buffer and centrifuged at 186,000 ***g*** for 30 min at 4°C in a TLA 55 rotor (Beckman Coulter, IN, USA). Pellets were resuspended in 1× Laemmli sample buffer, separated by SDS-PAGE, and stained with Coomassie Brilliant Blue. Gels were imaged on an Epson scanner (CA, USA) and band intensities were quantified using the Image Studio software (LI-COR Biosciences, Cambridge, UK).

### Statistical analysis and software

Datasets were tested for normality (Shapiro–Wilk) and equal variance. Statistical significance was analyzed using two-tailed unpaired Student's *t*-test, one-way ANOVA, or two-way ANOVA for independent samples. Tukey's multiple comparison test was applied to correct for multiple comparisons. GraphPad Prism8 (GraphPad, CA, USA) or R (version 3.6.0) software was used for this analysis. *P*-values are indicated by asterisks: *P<0.05, **P<0.01, ***P<0.001, and ****P≤0.0001. Results are shown as means±s.e.m. Immunoblot signals were quantified using the software Image Studio™ (LI-COR Biosciences). For FLIM-FRET, data was analyzed and plotted using R (Version 1.3.1093) with the ggplot2 package using Spearman rank correlation test.

## Supplementary Material

Supplementary information
