# Supplementary material for: Force-induced changes of α-catenin conformation stabilize vascular junctions independently of vinculin
Source: J Cell Sci. 2021 Dec 22;134(24):jcs259012. doi: 10.1242/jcs.259012 (PMC8729784; doi:10.1242/jcs.259012)
Supplement: Supplementary information [file joces-134-259012-s1.pdf]

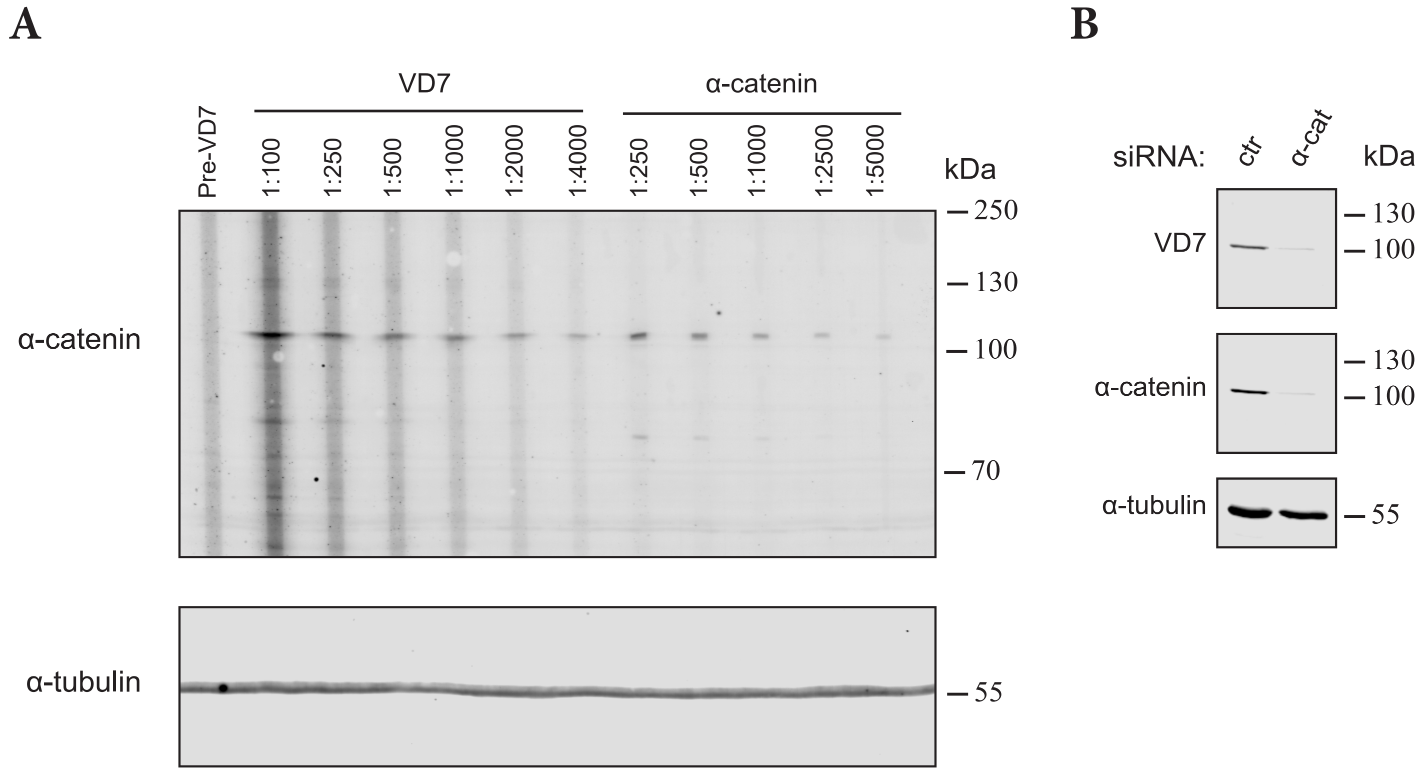

Fig. S1. VD7 antibody validation.
(A) Specificity of the VD7 antibody was tested on HUVEC lysates by western blot analysis. Preimmune-serum, VD7 immune serum or a commercial α-catenin antibody were tested at the indicated dilutions. (B) HUVECs were transfected with control siRNA or α-catenin–targeting siRNA for 72h. Total cell lysates were immunoblotted with affinity purified VD7 antibodies or commercial antibodies against α-catenin and α-tubulin (as indicated).

Journal of Cell Science • Supplementary information

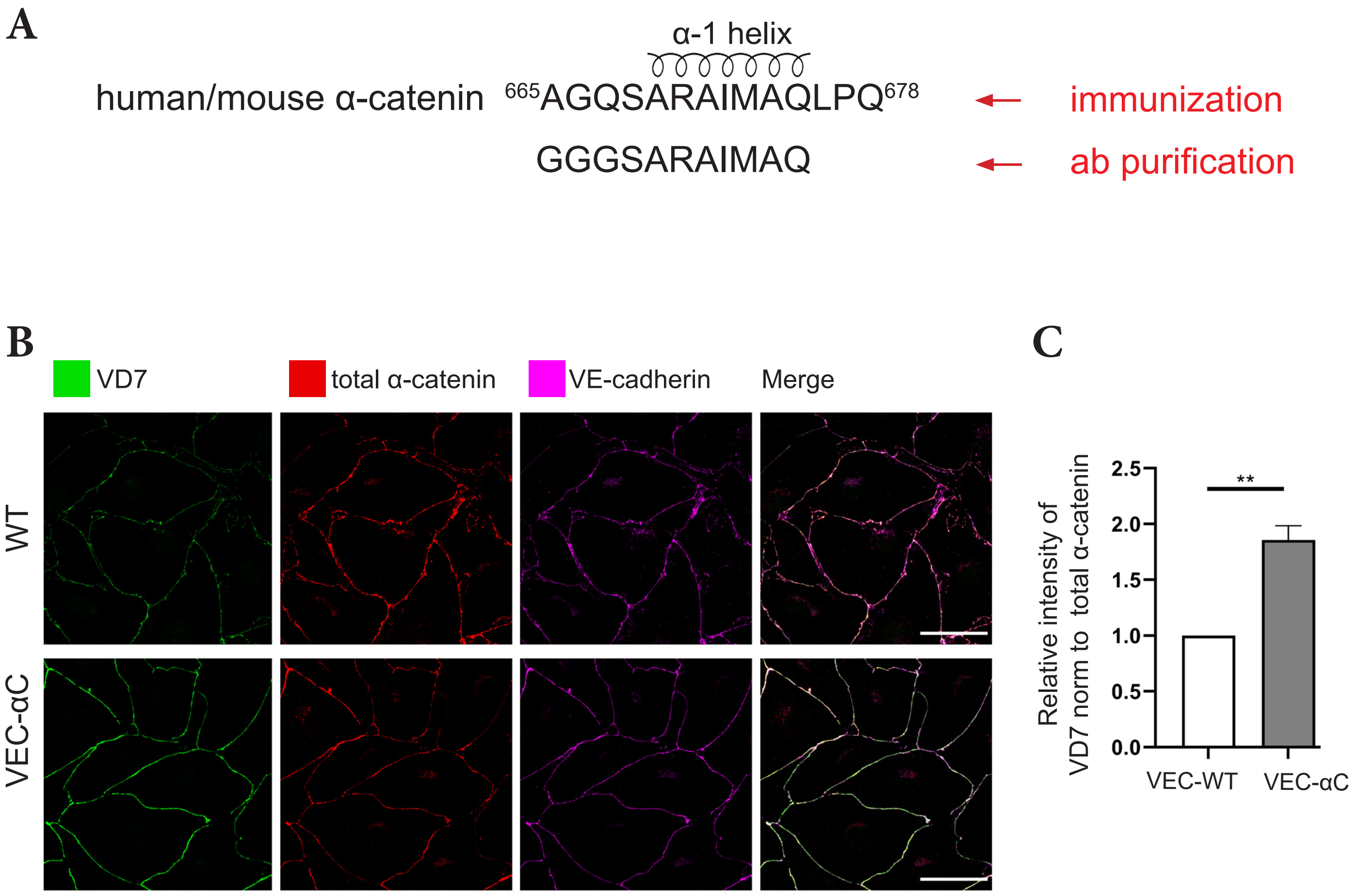

Fig. S2. Unfolding of the 7 aa α1-helix of α-catenin in junctional VEC-αC, as documented by antibodies restricted to this stretch of amino acids.
(A) Peptide sequence of α-catenin used for rabbit immunization (top) and for antibody purification (bottom). Note that both peptides share the 7 amino acids that form the α1-helix in α-catenin. (B) Confluent MDMVECs were fixed, permeabilized, and stained with the VD7 antibody restricted to the α1-helix, total α-catenin and VE-cadherin antibodies. (C) Quantification of the signal intensities of staining with the 7aa motif-specific VD7 antibodies relative to total α-catenin signal intensities as shown in (B) (n=3 independent experiments). Bars (B): 25µm. Statistical significance was analyzed using the unpaired two-tailed Student's t-test (C). Results are shown as means ±SEM. **, $P \le 0.01$.

Journal of Cell Science • Supplementary information

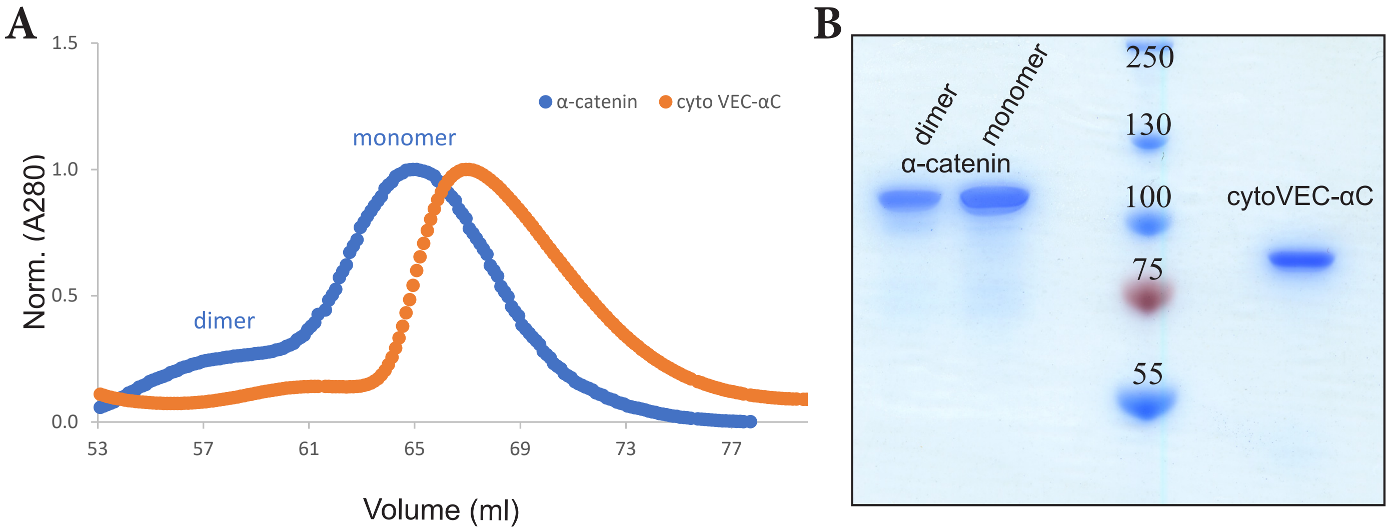

Fig. S3. Purification of recombinant α-catenin and the cytoplasmic tail of VEC-αC.
(A) Size exclusion chromatography of recombinant α-catenin and the cytoplasmic tail of VEC-αC (cytoVEC-αC). (B) Coomassie-stained SDS-PAGE of isolated dimeric and monomeric α-catenin and cytoVEC-αC (data from one representative experiment of three independent experiments).

Journal of Cell Science • Supplementary information

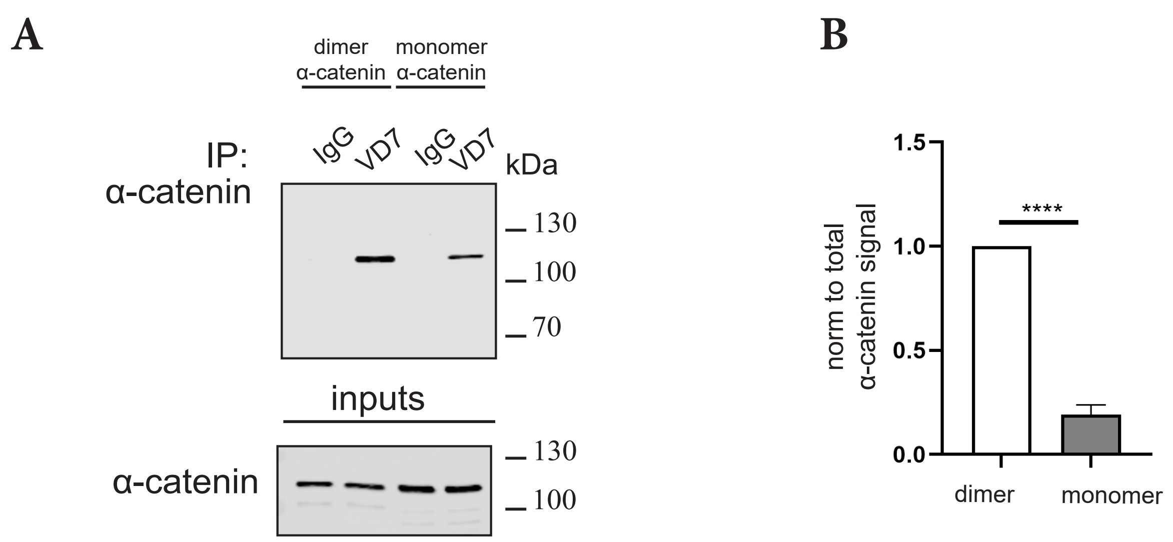

Fig. S4. The α1-helix is unfolded in dimeric α-catenin.
(A) α-catenin was precipitated from purified dimeric and monomeric α-catenin preparations using the VD7 antibody. Isotype-matched antibodies were used as a control. The immunoprecipitates were analyzed by SDS-PAGE and immunoblotted for total α-catenin. (B) Quantification of immunoprecipitated α-catenin dimers and monomers relative to total α-catenin input as shown in (A) (n=3 independent experiments). Statistical significance was analyzed using the unpaired two-tailed Student's t-test. Results are shown as means ±SEM. **** P ≤ 0.0001.

Table S1. Genotypes from intercrosses of VEC-αC_ΔVBD and VEC-αC_swapVBD mice

Mating	no. of litters	no. of offspring	+/VEC-αC_ΔVBD	VEC-αC_ΔVBD /VEC-αC_ΔVBD	% of VEC-αC_ΔVBD /VEC-αC_ΔVBD (% of expected)
+/VEC-αC_ΔVBD x VEC-αC_ΔVBD/ VEC-αC_ΔVBD	97	239	199	40	17 (34)

Mating	no. of litters	no. of offspring	+/VEC-αC_swapVBD	VEC-αC_swapVBD /VEC-αC_swapVBD	% of VEC-αC_swapVBD /VEC-αC_swapVBD (% of expected)
+/VEC-αC_swapVBD x VEC-αC_swapVBD/VEC-αC_swapVBD	39	152	104	48	32 (64)
